# Electrochemical Evaluation of Selenium (IV) Removal from Its Aqueous Solutions by Unmodified and Modified Graphene Oxide

**DOI:** 10.3390/molecules24061063

**Published:** 2019-03-18

**Authors:** Zuzana Koudelkova, Zuzana Bytesnikova, Kledi Xhaxhiu, Monika Kremplova, David Hynek, Vojtech Adam, Lukas Richtera

**Affiliations:** 1 Department of Chemistry and Biochemistry, Mendel University in Brno, Zemedelska 1, CZ-61300 Brno, Czech Republic; z.koudelkova@seznam.cz (Z.K.); zuzka.bytesnikova@gmail.com (Z.B.); mkremplova@volny.cz (M.K.); d.hynek@email.cz (D.H.); vojtech.adam@mendelu.cz (V.A.); 2 Central European Institute of Technology, Brno University of Technology, Purkyňova 656/123, CZ-61200 Brno, Czech Republic; 3 Department of Chemistry, Faculty of Natural Sciences, University of Tirana, Blv. Zog I, No. 2/1, 1001 Tirana, Albania; kledix@gmail.com

**Keywords:** selenium removal, graphene oxide, water purification, electrochemistry, differential pulse cathodic stripping voltammetry

## Abstract

The removal of selenium from superficial and waste water is a worldwide problem. The maximum limit according to the World Health Organization (WHO) for the selenium in the water is set at a concentration of 10 μg/L. Carbon based adsorbents have attracted much attention and recently demonstrated promising performance in removal of selenium. In this work, several materials (iron oxide based microparticles and graphene oxides materials) and their composites were prepared to remove Se(IV) from water. The graphene oxides were prepared according to the simplified Hummer’s method. In addition, the effect of pH, contact time and initial Se(IV) concentration was tested. An electrochemical method such as the differential pulse cathodic stripping voltammetry was used to determine the residual selenium concentration. From the experimental data, Langmuir adsorption model was used to calculate the maximum adsorption capacity. Graphene oxide particles modified by iron oxide based microparticles was the most promising material for the removal of Se(IV) from its aqueous solution at pH 2.0. Its adsorption efficiency reached more than 90% for a solution with given Se(IV) concentration, meanwhile its maximal recorded adsorption capacity was 18.69 mg/g.

## 1. Introduction

Increasing concentrations of metals and metalloids in the environment still represent a threat to biota, whereas bioremediation and/or physico-chemical cleaning of various water sources are challenging issues [1,2,3]. Selenium belongs to essential metalloids, however, its unique features lay within a very thin line between essentiality and toxicity [4,5]. In nature, selenium occurs in both organic and inorganic forms. The organic forms of selenium include amino acids selenomethionine and selenocysteine. Inorganic forms include these oxidation states: Selenite (SeO_3_^2−^), selenide (Se^2−^), selenate (SeO_4_^2−^), and elementary selenium (Se^0^) [6,7]. In the human body, selenium, with respect to its oxidation state and form, plays an important role in numerous biological processes and biochemical pathways, such as maintaining antioxidant homeostasis, formation of thyroid hormones, DNA synthesis, and reproductive cycles [6,8]. Selenium is also critical for muscles, where it improves their endurance and slows the aging process [5,9].

In the recent decades, the interest in selenium and its application in molecular, genetic, and health areas have grown [10,11]. With the wider use of selenium, the fear of its adverse environmental impact has increased [12]. The result was the inclusion of selenium into the set of environmental pollution indicators to be monitored according to the guidelines for drinking-water quality [13].

Selenium in the environment can be mobilized to enter the food chain through fish or plants [12,14,15]. It can cause short- or long-term damages to human health. Short-term health effects include the hair loss, nail deterioration, and damage of the peripheral nervous system, fatigue, and irritability. Prolonged exposure to high concentrations of selenium can cause kidney and liver dysfunction, as well as damages to the nervous and circulatory system [16,17,18]. An excess of selenium exhibit similar negative health effects on cattle and leads to reduction of their production [19]. The most common forms of selenium in water and in soils are selenite and selenate. It is assumed that in animal studies, selenite is slightly more toxic compared to selenate [20]. The occurrence and stability of selenium (different oxidative forms) largely depend on pH and Eh (oxidation-reduction potential) [20].

The main aim of this study is to find a suitable material for the effective removal of Se(IV) from the aquatic environment using electrochemical procedures. The advantage of electrochemical analysis is their speed, reliability, and above all, their simplicity. Carbon based adsorbents are auspicious materials for the removal of heavy metals and metalloids from the aqueous solutions. Carbon materials are also suitable to use as bioactive elements for biosensors. Such biosensors, for example, functionalized graphene oxide (GO), are widely used in biomedical applications [21,22]. It can be considered that GO is a suitable candidate due to its unique properties to act as an adsorbent for the removal of selenium. GO is a two dimensional (2D) carbon based material with high surface area containing a large number of functional groups (hydroxyl, carboxyl, and epoxy) on its surface [23,24,25]. Graphene oxide has high adsorption capacity, strong affinity, and can be modified easily [26]. The affinity of the GO towards the metal ions and semiconductors, GO could be part of a sensor for direct detection of selenium ions in the environment [23]. The aim of the study is also to compare different fractions of the same material that differ in size and to find out which has greater adsorption efficiency. The study assumes differences in different GO sizes, because the larger GO particles should have more functional groups than larger GO particles.

It has also been demonstrated that magnetic particles and their various modifications are used to remove metals and metals from water. The advantages of magnetic particles include chemical and physical stability, price, biocompatibility, and environmental acceptability [27,28]. They are also used to create biosensors for electrochemical analyzes. In addition, magnetic composites increase this yield, sensitivity, and specificity by combining different adsorption mechanisms [29,30]. Interaction of GO with iron-based materials has a synergistic effect of sorption capacity. Previous studies have shown excellent performance of GO and iron-based composites to remove pollutants from large volumes of sewage [31,32].

## 2. Experimental

### 2.1. Chemicals

Chemicals used in this study were purchased from Sigma-Aldrich (St. Louis, MO, USA) in American Chemical Society (ACS) purity. The deionized water was prepared using reverse osmosis equipment Aqual 25 (Tisnov, Czech Republic). The deionized water was further purified by using the apparatus MilliQ Direct QUV, equipped with the UV lamp (Aqua Osmotic, Tisnov, Czech Republic). The resistance was 18.2 MΩ. The pH was measured using pH meter WTW inoLab (Weilheim, Germany).

### 2.2. Preparation of Large Area Graphene Oxide

Graphite oxide was used as a starting material for GO synthesis and was prepared by chemical oxidation of 5.0 g graphite flakes (100 mesh, ≥75% min) in a mixture of concentrated H_2_SO_4_ (670 mL, 95.0–98.0%) and 30.0 g KMnO_4_ (purity >99%) according to the simplified Hummer’s method [33] described. GO was prepared according to the same procedure, as already reported [34].

### 2.3. Preparation of Small Area Graphene Oxide

Supernatant was taken from the last centrifugation step (see in Preparation of large area GO) and re-centrifuged. Very fine isolated GO was subsequently washed several times with Milli-Q water to achieve a constant pH (4–5). Separation of the GO solution was done exclusively by centrifugation (25,000 RCF, from 30 to 90 min). The preceding product was obtained by successive washing with water to form a stable colloidal solution of large area graphene oxide (GOH). Small area graphene oxide GOJ was diluted to 1 mg/mL concentration; the resulting pH was 3.8.

### 2.4. Preparation of Iron Oxide Based Microparticles

Amount of 7.48 g of Fe(NO_3_)_3_·9H_2_O was dissolved in 400 mL water, then 1.0 g of Na(BH)_4_ was added in 50 mL of 3.5% NH_3_. The reagents were mixed together by stirring and heating at 100 °C for 2 h, followed by stirring for 24 h. After the interaction the microparticles (MPs) were separated by centrifugation and washed three times with Milli-Q water. MPs were diluted to a concentration of 1 mg/mL, their pH were 7.1.

### 2.5. Preparation of Large Area Graphene Oxide Modified by Iron Oxide Based Microparticles

Volume of 5 mL of MPs were separated, washed three times with Milli-Q water and diluted to initial volume. 5 mL of large area GO was added to this sample, the resulting concentration of large area graphene oxide modified by iron oxide based microparticles (MGOH) was 1 mg/mL with pH 5.6. This mixture was stirred for 24 h.

### 2.6. Preparation of Small Area Graphene Oxide Particles (More Exfoliated) Modified by Iron Oxide Based Microparticles

Volume of 5 mL of MPs were separated, washed three times with Milli-Q water and diluted to initial volume. 5 mL of smaller area GO with smaller particles was added to this sample, the resulting concentration of small area graphene oxide modified by iron oxide based microparticles (MGOJ) was 1 mg/mL with pH 5.8. This mixture was stirred for 24 h.

### 2.7. Adsorption Experiments

**Figure 1:**
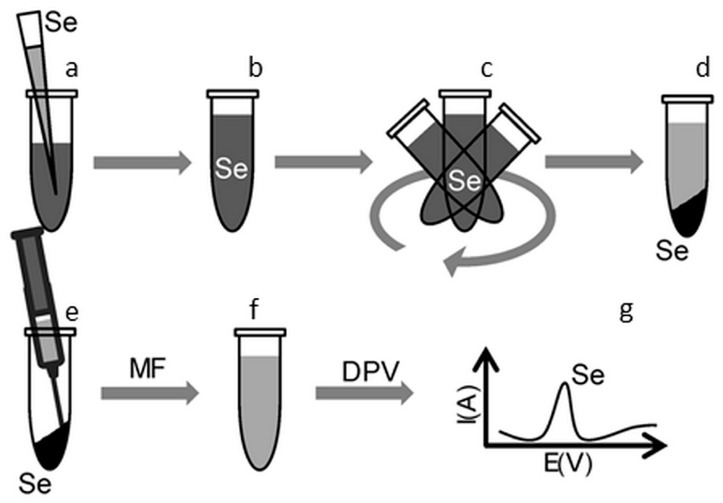
The scheme of selenium removal from aqueous solution using different types of adsorbents: (**a**) Adding Se(IV) to a carbon based adsorbent; (**b**) mixture of adsorbent and standard solution of sodium selenite; (**c**) shaking and mixing of the sample; (**d**) centrifugation; (**e**) removal of supernatant; (**f**) filtration using membrane filter (MF) 0.45 µm; and (**g**) electrochemical determination of Se(IV) by differential pulse cathodic stripping voltammetry (DPCSV).

Preparation of samples to remove Se(IV) from the aqueous solution was performed as follows: 250 µL of Milli-Q water and 500 µL of standard solution of sodium selenite (with different concentration of Se) was added to 250 µL of carbon based materials or MPs. These samples were shaken for a chosen time period using Thermomixer Comfort (Eppendorf, Germany). After the interaction time, the solid part of the samples was separated by centrifugation. The supernatant was removed using a syringe and filtered through the membrane filter with pore size 0.45 µm (BRAND^®^ accu-jet^®^, Sigma-Aldrich) to separate potential non-sedimenting microparticles. The concentration of Se(IV) was determined by potentiometric technique differential pulse cathodic stripping voltammetry (DPCSV) in purified supernatant, and using this information, the concentration of Se(IV) adsorbed on the adsorbent surface was calculated (Figure 1).

The adsorption efficiencies were calculated according to the formula:

(1) $$A_{e}=100\%-\frac{c_{D}}{c_{V}}\times 100\%,$$

*A_e_* is adsorption efficiency, *c_D_* is the detected concentration of metal in the filtrate and *c_V_* is the concentration of the bounded metal [35].

### 2.8. Electrochemical Determination of Se(IV) Using Differential Pulse Cathodic Stripping Voltammetry

Determination of selenium by differential pulse cathodic stripping voltammetry (DPCSV) was performed with 797 VA Computrace instrument connected to 889 IC Sample Center (Metrohm, Switzerland), using three electrodes connections. The hanging mercury drop electrode (HMDE) with a drop area of 0.4 mm^2^ was used as the working electrode. An Ag/AgCl/3 M KCl was the reference electrode and platinum wire served as an auxiliary electrode. The analyzed samples were deoxygenated prior to measurements by purging with argon (99.999%). As a supporting electrolyte a mixture of 0.13 M ammonium sulphate, 0.12 mM copper sulphate and sulfuric acid (to adjust pH to 2.2) was used. The supporting electrolyte was exchanged after each measurement. The parameters of the measurement were as follows: Deoxygenating with argon 120 s, initial potential of −0.4 V, end potential −0.9 V, deposition time 200 s, deposition potential −0.6 V, time interval 0.05 s, voltage step 6 mV, pulse amplitude 30 mV, volume of sample 20 µL, and volume of electrochemistry cell 2 mL (20 μL of sample and 1980 μL electrolyte). The analytes were measured three times to check the efficiency of the modified electrode.

### 2.9. Characterterization of Size and Zeta Potential of the Adsorption Material Subsection

The zeta potential and size of particles were measured using the Zetasizer Nano ZS instrument (Malvern Instrument Ltd., Worcestershire, UK). The parameters of particle size measurements were as follows: Refraction index of the dispersive phase of 3.00 and 1.333 for the dispersive environment, adsorption coefficient 10^−3^, temperature 25 °C, equilibration time 120 s, and measurement angle of 173° backscatter. For measurement, disposable cuvettes types ZEN 0040 were used, containing 50 µL of sample. The zeta potential measuring parameters such as temperature and equilibrating time were the same as in particle size measurements. Calculations considered the diminishing of particles concentration based Smoluchowsky model, with parameters F(κa) of 1.50. For measurement, disposable cuvettes type DTS1070, were used. The measurements were performed under the automatic setting of attenuation and voltage selection. All measurements were in triplicate.

### 2.10. Scanning Electron Microscopy

Scanning electron microscopy (SEM) method was used to characterize the GO and composite materials structure. The device settings were used, as in the previous study [34].

## 3. Results and Discussion

### 3.1. Electrochemical Determination of Se(IV) Using Differential Pulse Cathodic Stripping Voltammetry

Electrochemical analysis is used to characterize the applicability of various carbon materials and materials modified by the MP. Carbon based materials can be used as part of biosensors, which would be suitable, for example, for selenium detection.

**Figure 2:**
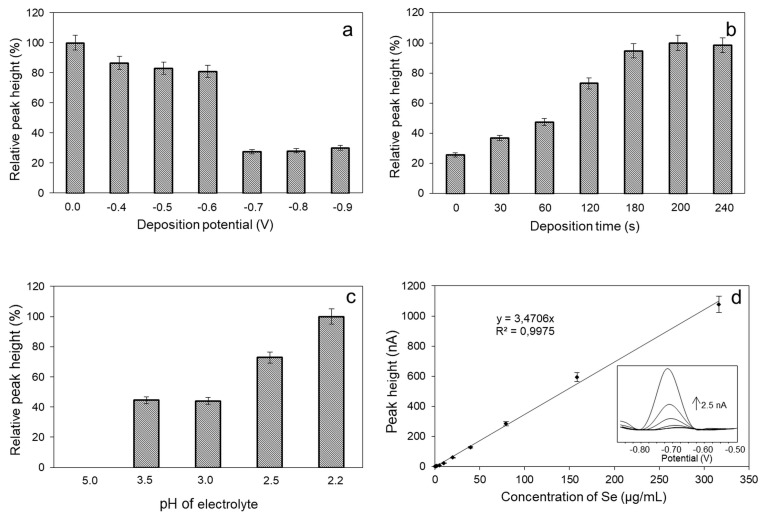
Optimization of the parameters of the method for Se(IV) determination: (**a**) The influence of deposition potential on the relative peak height of Se(IV); (**b**) the dependence of relative peak height on the deposition time; (**c**) the dependence of relative peak height on the supporting electrolyte pH (5.0, 3.5, 3.0, 2.5, and 2.2); and (**d**) calibration curve of Se(IV) for the concentration range 0.2–318 µg/mL measured by DPCSV in 0.13 M ammonium sulphate, in the presence of 0.12 mM copper sulphate and sulfuric acid (pH adjusted to 2.2) playing the role of supporting electrolytes.

For an automatic determination of Se(IV), DPCSV was used due to its excellent sensitivity for the detection of metal ions, metalloids and other electrochemically active substances [36,37]. A reduction peak was observed around −0.7 V which might be due to the reduction of Se(IV). This peak can be used for electroanalytical quantification of the analyte. To obtain a high sensitivity of the method, various individual parameters were optimized. The deposition potential within the range of 0.0 to −0.9 V was the first optimized parameter. Figure 2a shows that the highest and the most stable electrochemical signal was observed at a deposition potential of −0.6 V, thus this value was used for all other electrochemical measurements. The deposition time associated with the deposition potential was the next optimized parameter. Figure 2b shows the increasing electrochemical signal of Se(IV) with the increasing of the deposition time. The highest electrochemical response was achieved for a deposition time of 200 s. The pH of the supporting electrolyte is closely related to the sensitivity of electrochemical determination of metals and transition elements. In the case of Se(IV) determination, pH values of 5.0, 3.5, 3.0, 2.5, and 2.2 were tested. The pH value of 2.2 provides the most stable electrochemical signal, therefore, it was chosen for all further Se(IV) measurements (Figure 2c). The obtained results are in good agreement with the previously published ones [16]. After setting the optimal conditions of the method, the calibration curve for Se(IV) determination within the concentration range from 0.2 to 318 µg/mL was recorded. The plotted peak height vs. Se concentration displayed a linear trend expressed by the equation y = 3.4706x with the fitting coefficient of R^2^ = 0.9975, limit of detection of 0.02 µg/mL and limit of quantification of 0.07 µg/mL. The characteristic peak of Se(IV) was recorded for the potential value of −0.715 V (Figure 2d).

### 3.2. Characterization of Graphene Oxide Based Materials and Microparticles

**Figure 3:**
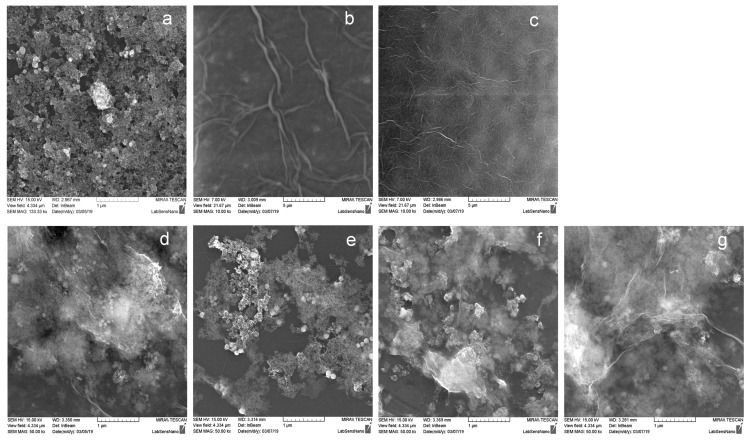
Scanning electron microscopy (SEM) characterization of materials: (**a**) Structure of microparticles (MPs); (**b**) structure of large area graphene oxide (GOH) with bigger wrinkles; (**c**) structure of small area graphene oxide (GOJ) with little and finer wrinkles; (**d**) GOH layered structure wrapping MPs; (**e**) GOJ layered structure wrapping MPs; and (**f**) large area graphene oxide modified by iron oxide based microparticles (MGOH) composite after the regeneration process; MGOH composite with Se(IV) bound (**g**).

**Figure 4:**
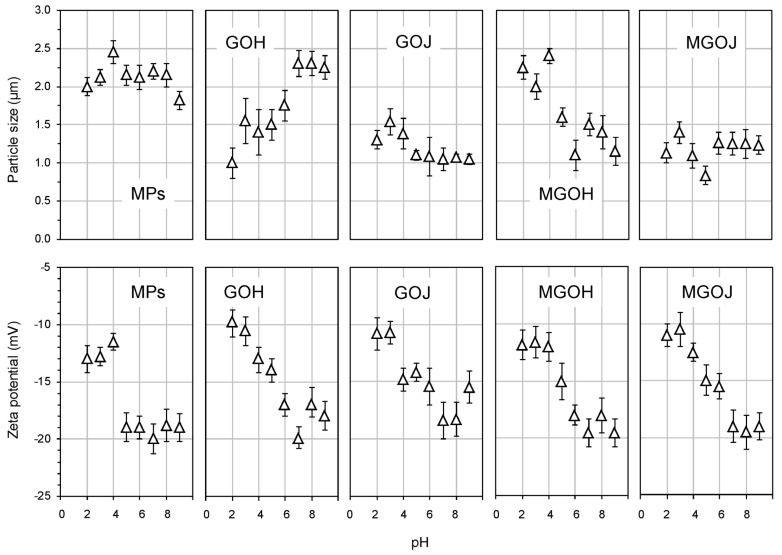
Comparison of the mean size and zeta potential of the individual materials employed in different pH values.

The illustrative SEM image (Figure 3) shows the presence of GO layer decorated with MPs flakes. Graphene oxide synthesized for the scope of this study had similar characteristics as the previously reported GO [38]. In comparison to the Fu’s experiment [16], our GO has finer structure thanks to its washing with HCl and effective centrifuging. The preparation process of our MPs is simpler and takes place under more gentle conditions. The effect of pH on the mean particle size and zeta potential were studied for the prepared materials. The obtained results were plotted in the graphs shown in Figure 4 with the respective standard deviations of the measured data. The size of individual materials is about 2.5 nm. As can be seen from zeta potential measurements, carbon materials are particularly stable in alkaline areas and observed zeta potential values are similar in nature to the published study [39]. According to this study, the influence of pH on the behavior of GO layers is well visible at low pHs, where highly protonated carboxyl groups are present, resulting in less hydrophilic structures [39]. Table 1 gives an overview of used materials and their natural pH values. The natural pH values for different composite materials fall between the pH values of their constituents (MPs, GOH, and GOJ). The SEM image shown in Figure 3 reveals the well preserved GO layered structure with the MPs wrapped in them.

### 3.3. Interaction of Graphene Oxide Based Materials and Microparticles with Se(IV)

Materials based on GO and MPs exhibit adsorption abilities toward heavy metals and can be used, e.g., for decontamination of surface water and waste water polluted by these metals and metalloids [23,35]. In this experiment, five different materials (MPs, GOH, GOJ, MGOH, and MGOJ) were tested for their ability to adsorb Se(IV) from its aqueous solution. To prove these properties, the interaction time of these materials using a standard solution of sodium selenite was studied for different contact times: 1, 5, 10, 15, 30, 60, 90, and 120 min. The interaction of GO based materials containing MPs with Se(IV) and the influence of pH on this interaction were investigated at room temperature. Although it is beyond the scope of this study, on the basis of other similar studies, it is assumed that the regeneration and reusability of modified GO would be possible [40,41,42].

**Figure 5:**
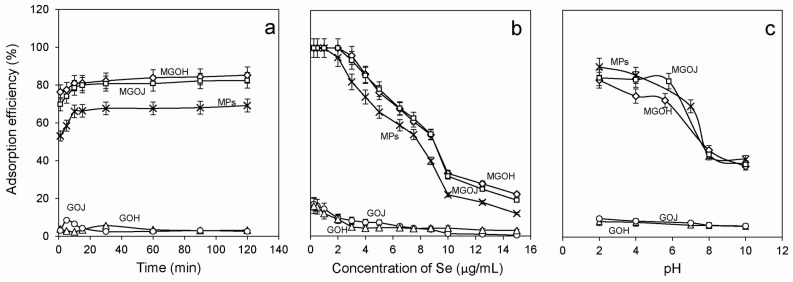
(**a**) The variation of Se(IV) adsorption efficiency from the interaction time for unmodified (GOH, GOJ, MPs) and the modified materials (MGOH and MGOJ) in a solution of initial Se(IV) concentration of 5 µg/mL; (**b**) comparison of the effect of Se(IV) concentration on the adsorption efficiency of different materials considered (MPs, GOH, GOJ, MGOH, MGOJ); and (**c**) the influence of the pH values of different carbon materials used on the adsorption efficiency of Se(IV) from its aqueous solution containing 5 µg/mL.

The graphs in Figure 5a show the adsorption efficiency of Se(IV) for unmodified (GOH and GOJ) and modified materials by MPs at natural pH. 10% adsorption efficiency was achieved when using unmodified materials (GOH, GOJ). Due to the low adsorption properties of the unmodified materials toward Se(IV), the modification of these adsorbents by MPs was performed. For the considered adsorbents (MPs, MGOH, and MGOJ) the same trend is observed—with the increase of contact time the efficiency of adsorption of Se(IV) from an aqueous solution increases. The adsorption efficiency of Se(IV) reached more than 80% after 60 min of contact time. The highest adsorption was reached when using MGOH and MGOJ materials. Figure 5b shows the effect of adsorption efficiency of carbon materials in the natural pH with respect to different concentrations of Se(IV). The composites materials (GP modified by MPs) displayed better results compared to the unmodified materials. Figure 5c shows the adsorption evolution of Se(IV) as a function of materials pH. The maximum adsorption was achieved at pH 2.0, this finding is also confirmed by other studies [16]. For further comparison, we chose small area graphene oxide in pH 2 (MGOJ-2pH) and large area graphene oxide at pH 2 (MGOJ-2pH). The anticipated mechanism of selenium adsorption on the GO surface is explained in previous studies [5,16], where the best results were achieved by the materials modified by magnetic nanoparticles. In this study, composite iron oxide based microparticles with carbon materials were used. Micro- and nano-particles among themselves may differ, especially due to their structure and easiness of separation [43].

**Figure 6:**
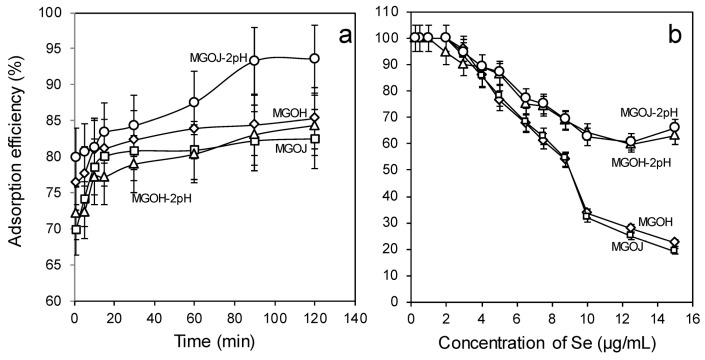
(**a**) The relative adsorption efficiency of Se(IV) from its solution with a concentration of 5 µg/mL versus the contact time of 1, 5, 10, 15, 30, 60, 90, and 120 min for MPs, MGOH, MGOJ, MGOH-2pH, MGOJ-2pH; and (**b**) The effect of Se(IV) concentration on the adsorption efficiency for different materials MGOH, MGOJ, MGOH-2pH, and MGOJ-2pH.

Based on the highest adsorption efficiencies obtained for MGOJ and MGOH at pH 2.0 (Figure 5c), a comparison of the adsorption efficiency of these materials versus the contact time for the pH 2 and their natural pH is depicted in Figure 6a. MGOJ adsorbs better at pH 2 compared to MGOH, where its Se(IV) adsorption efficiency reached over 93% within 90 min of contact time, meanwhile MGOH shows a better adsorption behavior than MGOJ at its natural pH.

In Figure 6b, the comparison of the Se(IV) adsorption efficiency of MGOH and MGOJ at natural and at pH 2.0 with respect to various Se(IV) concentrations is shown. It is well observable from it that a decrease of the pH value from the natural to the pH 2 increases the Se(IV) adsorption efficiency with the increase of its concentration. The highest adsorption efficiency was achieved for MGOJ at pH 2.0. At this pH, for Se(IV) concentrations of 9–15 µg/mL MGOJ reveals an adsorption efficiency higher than 65%, meanwhile for Se(IV) concentrations of 4 µg/mL and below it, relative adsorption efficiencies varying from 90% to 100% are possible.

Table 2 contains an overview of relative adsorption efficiencies for all materials used to remove Se(IV) from its solution of 5 µg/mL. Based on it, the best way to remove Se(IV) from water was obtained when using MGOH at 2pH, as confirmed by the respective graph (Figure 5a).

### 3.4. Adsorption Characteristics

To evaluate the adsorption behavior of Se(IV) on the selected materials, the adsorbed amount was calculated based on the following equation:

(2) $$q=\left(C_{0}-C_{e}\right)\times \frac{V}{m},$$

where *C_0_* represent the initial and *C_e_* equilibrium concentrations, *V* is volume of standard solution and *m* represents the mass of adsorbent. For this characterization, and Se(IV) solutions with concentrations varying from 3 to 15 µg/mL were used. They were placed in contact with the selected modified and unmodified carbon materials for 2 h.

Based on the obtained absorption data, the linearized Langmuir’s isotherms were calculated according to the equation:

(3) $$\frac{1}{q_{e}}=\frac{1}{q_{max}bC_{e}}+\frac{1}{q_{max}},$$

where *q_e_* corresponds to the amount of analyte (Se(IV)) adsorbed at equilibrium and *b* represents the Langmuir constant.

**Figure 7:**
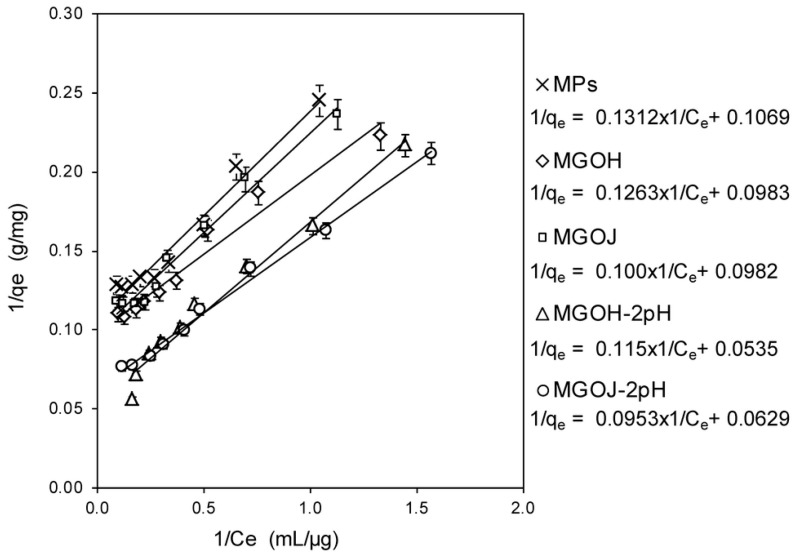
The Langmuir isotherms of the adsorption behavior of Se(IV) on MPs, MGOH, MGOJ, MGOH-2pH, and MGOJ-2pH.

The calculated values for the maximum adsorption capacity (*q_max_*) of Se(IV) from MPs, MGOH, MGOJ, MGOH-2pH, and MGOJ-2pH are shown in Figure 7, meanwhile the fitted values for the parameters of Langmuir’s isotherm model are listed in Table 3. The obtained data revealed MGOH at pH 2.0 as the best material for Se(IV) adsorption from its aqueous solution. The best adsorption capacity was reached by GO materials modified with MPs at pH 2.0, which is in full compliance to further studies mentioning that a decreased pH is better for the successful removal of selenium from its aqueous solutions [35,44,45,46].

## 4. Conclusions

This study sheds light on removal of Se(IV) from its aqueous solutions using different composite materials based on GO and their modifications with MPs. The composite material MGOH at pH 2 was identified as the best adsorbent for the effective removal of Se(IV) from its aqueous solution. Its adsorption efficiency was over 93% in a of Se(IV) solution with initial concentration of 5 µg/mL within 90 min of contact time. This composite bears several advantages such as the easiness and low cost production in large quantities, making it particularly suitable for purifying contaminated and/or waste water. These types of adsorption materials and their results can serve as preliminary data for creating a sensor for selenium determination in the aquatic environment. Our experimental data proved the distinguished properties of MGOH-2pH composite as a suitable and promising sorbent for the successful removal of Se(IV).

## Figures and Tables

**Table 1:** The natural pH values of the employed materials microparticles (MPs), large area graphene oxide (GOH), small area graphene oxide (GOJ), large area graphene oxide modified by iron oxide based microparticles (MGOH), and small area graphene oxide modified by iron oxide based microparticles (MGOJ).

Material	Natural pH
MPs	7.1
GOH	3.5
GOJ	3.8
MGOH	5.6
MGOJ	5.8

**Table 2:** The relative values of the adsorption efficiency from a solution with Se(IV) concentration of 5 µg/mL for MPs, GOH, GOJ, MGOH, MGOJ, MGOH-2pH, and MGOJ-2pH.

Material	Relative Adsorption Efficiency (%)
MPs	66.20
GOH	4.85
GOJ	7.18
MGOH	80.14
MGOJ	81.79
MGOH-2pH	91.05
MGOJ-2pH	90.23

**Table 3:** Langmuir adsorption isotherm’s parameters for the removal of Se(VI) from its aqueous solution.

Material	*b*	*q_max_* (mg/g)
MPs	0.82	9.35
MGOH	0.78	10.17
MGOJ	0.98	10.18
MGOH-2pH	0.47	18.69
MGOJ-2pH	0.66	15.90
